# Supplementary material for: Identification of photoperiod-induced specific miRNAs in the adrenal glands of Sunite sheep (Ovis aries)
Source: Front Vet Sci. 2022 Jul 22;9:888207. doi: 10.3389/fvets.2022.888207 (PMC9354845; doi:10.3389/fvets.2022.888207)
Supplement: Supplementary file 1 [file Data_Sheet_1.ZIP › Supplementary file/miRDeep2 file.zip!/miRDeep2 file/Know_Structure/LP42A3/oar-mir-21.pdf]

miRBase precursor : oar-mir-21
Total read count : 169281
oar-miR-21 read count : 169268
remaining reads : 13

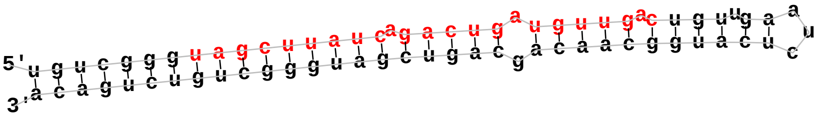

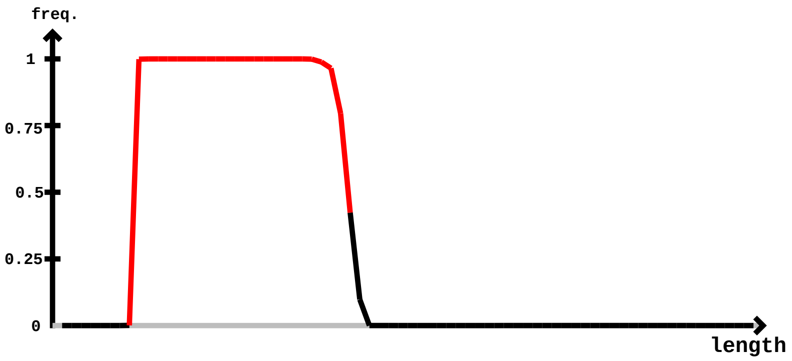

oar-miR-21

5'- ugucggguagcuuaucagacugauguugacuguugaaucucauggcaacagcagucgaugggcugucugaca -3'	exp		
((((((((((((((((.(((((.(((((.((((.((...))))))))))).))))))))))))))))))))	reads	mm	sample
......guagcuuaucagacugaugu...	7	0	seq
......guagcuuaucagacugauguu..	4	0	seq
......guagcuuaucagacugauguug...	61	0	seq
......guagcuuaucagacugauguuga..	29	0	seq
......guagcuuaucagacugauguugac...	8	0	seq
......guagcuuaucagacugauguugacu..	7	0	seq
.......uagcuuaucagacugau...	9	0	seq
.......uagcuuaucagacugaug..	149	0	seq
.......uagcuuaucagacugaugu...	1821	0	seq
.......uagcuuaucagacugauguu..	3921	0	seq
.......uagcuuaucagacugauguug...	28552	0	seq
.......uagcuuaucagacugauguuga..	62974	0	seq
.......uagcuuaucagacugauguugac...	55011	0	seq
.......uagcuuaucagacugauguugacu..	16520	0	seq
.......uagcuuaucagacugauguugacug.......................................	1	0	seq
.......uagcuuaucagacugauguugacugu......................................	6	0	seq
.......uagcuuaucagacugauguugacuguu.....................................	5	0	seq
.......uagcuuaucagacugauguugacuguugaau..................................	4	0	seq
........agcuuaucagacugaugu...	3	0	seq
........agcuuaucagacugauguu..	6	0	seq
........agcuuaucagacugauguug...	24	0	seq
........agcuuaucagacugauguuga..	56	0	seq
........agcuuaucagacugauguugac...	51	0	seq
........agcuuaucagacugauguugacu..	21	0	seq
.........gcuuaucagacugauguug...	2	0	seq
.........gcuuaucagacugauguuga..	11	0	seq
.........gcuuaucagacugauguugac...	5	0	seq
.........gcuuaucagacugauguugacug.......................................	1	0	seq
..........cuuaucagacugauguuga..	1	0	seq
...........uuaucagacugauguuga..	1	0	seq
............uaucagacugauguugacuguu.....................................	1	0	seq
....uggcaacagcagucgaugggcuguc.....	1	0	seq
....caacagcagucgaugggcug.......	1	0	seq
....caacagcagucgaugggcugu......	3	0	seq
....caacagcagucgaugggcuguc.....	4	0	seq

oar-miR-21

ugucggguagcuuaucagacugauguugacuguugaaucucauggcaacagcagucgaugggcugucugaca
